# Hepatoprotective activity of okra (*Abelmoschus esculentus* L.) in sodium nitrite-induced hepatotoxicity

**DOI:** 10.14202/vetworld.2020.1815-1821

**Published:** 2020-09-07

**Authors:** Sri Puji Astuti Wahyuningsih, Elma Sakinatus Sajidah, Baiq Naili Dewi Atika, Dwi Winarni, Manikya Pramudya

**Affiliations:** Department of Biology, Faculty of Science and Technology, Universitas Airlangga, Surabaya, Indonesia

**Keywords:** hepatoprotective, liver histology, okra pods methanol extract, sodium nitrite

## Abstract

**Background and Aim:**

For years, people have used sodium nitrite as a food preservative. This study determined the effect of okra (*Abelmoschus esculentus* L.) pod methanol extract (OPME) on mice with hepatotoxicity induced by sodium nitrite. The flavonoid and total phenolic levels, serum biochemistry, and liver histology were examined.

**Materials and Methods:**

Green okra pod extraction was performed using ethanol methanol solvent. Thirty adult male BALB/c mice (8-10 weeks, ~30 g) were divided into six groups: Normal control, negative control (sodium nitrite 50 mg/kg BW exposure), and treatment groups (sodium nitrite exposure and OPME at doses of 50, 100, 200, and 400 mg/kg BW). Subsequently, they were exposed to sodium nitrite and administered multiple doses of OPME for 19 days by gavage. After that, serum was used for biochemical evaluation, and liver histological analysis was performed. All data were statistically analyzed (α=0.05).

**Results:**

All doses of OPME reduced the levels of nitric oxide (NO), malondialdehyde (MDA), alanine aminotransferase (ALT), and aspartate aminotransferase (AST). In this research, both superoxide dismutase (SOD) and catalase (CAT) levels increased in all OPME-administered treatments. All doses also reduced necrotic cells, proportion of swollen cells, and inflammation in liver histological analysis. The results of this study showed that OPME exerted hepatoprotective effects by lowering MDA, NO, ALT, and AST levels. It also improved SOD and CAT levels and recovered damaged liver tissue to its normal state. The optimal dose of OPME was 50-100 mg/kg BW.

**Conclusion:**

OPME has potential as a natural hepatoprotective agent against sodium nitrite exposure.

## Introduction

Sodium nitrite is a chemical widely used for preserving food, especially cured meats [1]. However, it leads to continuous physiological changes. For example, according to Baek *et al*. [2], sodium nitrite can increase oxidative stress. Nitrites derived from sodium nitrite turn into nitrates and nitric oxide (NO) through oxidation and reduction reactions [3]. These reactions produce reactive oxygen species (ROS). Along with the formation of other harmful components such as nitrite chloride, these ROS cause cytotoxicity, and tissue damage.

NO is a reactive free radical that easily reacts with biomolecules. This oxidant can bind with superoxide to form peroxynitrite (ONOO^−^). The reaction between ONOO^−^ and polyunsaturated fatty acid (PUFA) rapidly enhances lipid peroxidation, which produces malondialdehyde (MDA). An increasing level of MDA leads to damage to the cell membrane, and widespread damage causes tissue and organ deterioration. In the liver, this can be seen by observing the number of necrotic cells and can also be identified using biochemical markers. The biochemical features of liver damage include the production of serum alanine aminotransferase (ALT) and serum aspartate aminotransferase (AST) enzymes [4]. Oxidative stress-induced by sodium nitrite can be overcome by antioxidants [5,6]. One plant that contains antioxidant compounds is okra (*Abelmoschus esculentus* L.). Specifically, this plant is known to have many antioxidants, such as flavonoids, quercetin, rutin, polysaccharides, pectin, and amino acids [7]. Luo *et al*. [8] reported that flavonoids in okra induce the activation of the nuclear factor-E2-related factor 2-activation response element (Nrf2-ARE). This process leads to the transcription of antioxidant genes such as superoxide dismutase (SOD) and catalase (CAT). SOD can bind superoxide and transform it into hydrogen peroxide, while CAT transforms hydrogen peroxide into oxygen and water [8].

In most studies of the antioxidative activity of okra, various oxidative agents such as CCl_4_ were analyzed [9,10]. However, to the best of our knowledge, no studies on the antioxidant activity of okra pod methanol extract (OPME) as a protective agent against sodium nitrite consumption have been performed. The purpose of this study was, thus, to determine the effect of OPME on the levels of NO, MDA, ALT, AST, SOD, and CAT, as well as the liver histology in *Mus musculus* with hepatotoxicity induced by sodium nitrite.

## Materials and Methods

### Ethical approval

All of the experiments involving animal care and the sacrifice procedure (using ketamine-xylazine) were approved by the Animal Care and Use Committee, Faculty of Veterinary Medicine, Airlangga University No. 2.KE.138.06.2019.

### Study period and location

This research was conducted for 7 months, from April to October 2019. The research was conducted in the Animal Laboratory and Molecular Biology Laboratory, Department of Biology, Airlangga University.

### Materials and chemicals

Sodium nitrite was purchased from E. Merck, Germany. In this study, Biotech^®^ MDA-586 spectrophotometric assay kit, Griess reagent, AST detection kit (ALAT FS Cat. No. 1 2701 9910 021), ALT detection kit (ASAT FS Cat. No. 1 2601 99 10 021), and SOD typed assay kit (E-BC-K022) were used. CAT activity was measured using the EnzyChrom™ CAT Assay Kit (ECAT-100). The other chemicals were hematoxylin-eosin, aquadest, neutral buffered formalin, ethanol, methanol, and xylene, along with paraffin pastilles purchased from E. Merck, Germany.

### Plant material and preparation

The fresh green okra pods (*A. esculentus* L.) were from an okra farm in Jember, Indonesia. The okra pods were cut and dried in the shade for 7 days. These dried pods (296 g) were then coarsely powdered and macerated in 96% methanol for 72 h, and the concentrates were collected every 24 h. The solvent was then removed at 80°C using a rotary evaporator. The evaporated extract (80 mL) was freeze-dried, and 52.3 g of total extract or OPME was obtained.

### The antioxidant activity of OPME

The antioxidant activity of OPME was measured using a total phenolic test and flavonoid level test. Total phenol was tested using Folin–Ciocalteu reagent. A total of 0.1 mL of sample extract at various concentrations was supplemented, with 0.1 mL of 50% Folin–Ciocalteu reagent. After 5 min, 2 mL of Na_2_CO_3_ solution (75 g/L) was applied. Then, the solution was placed in the dark for 30 min. Optical density (OD) values were measured at λ = 750 nm. The standard used was gallic acid. The flavonoid test was performed by mixing 0.5 mL of methanol with 0.5 mL of the sample. The next step was to add 50 mL of 10% aluminum chloride (AlCl_3_), 50 mL of potassium acetate, and 1.4 mL of distilled water. The OD value was measured at λ=415 nm. The standard used was quercetin.

### Animals

Male *M. musculus* BALB/c of approximately the same age (8-10 weeks), weighing 25-30 g, was obtained from the Faculty of Pharmacy, Airlangga University. The mice were kept at constant room temperature, humidity, and 12 h light-dark conditions. The animals were provided with food and water *ad libitum*.

### Administration of sodium nitrite and OPME

Mice were divided into six groups (KN, K-, P1, P2, P3, and P4) (n=5 animals/group). Group 1 (KN) was used as a standard control (using distilled water). Group 2 (K-) was administered sodium nitrite (50 mg/kg BW), and 2 h later was administered distilled water. Groups 3-6 (P1-P4) were administered sodium nitrite, and 2 h later were administered OPME at doses of 50, 100, 200, and 400 mg/kg BW, respectively. All administrations were performed orally. The sodium nitrite and OPME were administered once a day for 19 consecutive days (days 1-19).

### Serum isolation and organ collection

On the 20^th^ day, the animals were sacrificed using the retroorbital ketamine–xylazine injection technique. The blood was collected by cardiac puncture and allowed to clot at room temperature (28°C). The serum was separated by centrifugation (1308 g, 10 min). The liver was collected and fixed in neutral buffered formalin for histological examination.

### Examination of antioxidant enzymes (SOD and CAT levels)

The SOD activity was determined using a reaction system containing xanthine and xanthine oxidase. It produced O_2_. This O_2_ oxidizes hydroxylamine and forms nitrite, which appears as purplish-red color. A sample of 20 μL was added to the tube and mixed with 100 μL of reagent 1. After that, it was mixed with 20 μL of distilled water, 10 μL of reagent 2, 10 μL of reagent 3, and 10 μL of reagent 4. The sample was then vortexed and incubated for 40 min at 37°C. Then, 200 μL of the chromogenic agent was added, and the SOD level was read by determining the absorbance at 500 nm.

CAT activity was measured using the EnzyChrom™ CAT Assay Kit (ECAT-100). The protocol of this kit relies on the reaction of the enzyme in the presence of an optimal concentration of H_2_O_2_. The reagent prepared for the CAT test was detection reagent, in a reaction with 4.8 mM enzyme, and a positive control reagent. The detection reagent was made using 6120 μL of assay buffer, 1 μL of dye reagent, and 1 μL of HRP enzyme, while the enzyme reaction involved a combination of 5 μL of 3% H_2_O_2_ and 914 μL dH_2_O. Each sample required 1 μL of 4.8 mM H_2_O_2_ and 95 μL of assay buffer. In the 96-well plate used, there were wells for blanks and samples. Blanks contained 10 μL of assay buffer and 90 μL of enzyme reaction. After the 10 μL samples were added into the 96-well plate, it was mixed with 10 μL of positive control reagent, 10 μL of assay buffer, and 90 μL of enzyme reaction. Then, it was incubated for 30 min and supplemented with 100 μL of detection reagent. The CAT level was read by determining the absorbance at 570 nm.

### Examination of oxidant levels (NO and MDA levels)

Measurement of NO levels was performed using the Griess reaction colorimetric method. A serum sample of 50 μL was supplemented with 100 μL of Griess Reagent I and 100 μL of Griess Reagent II. The OD value was read at ƛ=540 nm using a ultraviolet/Vis microplate spectrophotometer. The NO level in the sample was determined by entering the OD value in the standard nitrite regression equation, which is y=0.242x + 0.083. Here, y is the serum OD and x is the level of nitrite (M).

The collected serum (80 μL) was supplemented with 4 μL of Probucol. A total of 256 μL of R1 solution and 150 μL of R2 were added to the serum. The mixture of the serum, R1, and R2 was incubated for 60 min at 45°C. Each tube was centrifuged at 10,000 g for 10 min. The supernatant was moved to a 96-well microplate, and the OD value was read at λ=586 nm. The MDA level (μM) was determined by the following regression equation: y=0.0045x + 0.0473, with y representing the OD value and x the MDA level (multiplied by the dilution factor).

### Evaluation of liver marker enzymes (ALT and AST levels)

A total of 50 μL of serum was mixed with reagents 1 and 2 available in the ALT test kit. For each sample, the first step was to combine the sample with 1000 μL of R1, followed by incubation for 5 min. Furthermore, 250 μL of R2 was added and the absorbance values were read at 1, 2, 3, and 4 min using a spectrophotometer at 340 nm.

The equation then determined the ALT value:


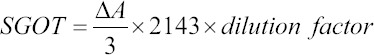


Where:

ΔA=The difference between the absorbance level at each measurement time [(A_1_-A_2_)+(A_2_-A_3_)+(A_3_-A_4_)]

A1 = Absorbance value at the 1^st^ min

A2 = Absorbance value at the 2^nd^ min

A3 = Absorbance value at the 3^rd^ min

A4 = Absorbance value at the 4^th^ min.

The sample (50 μL) was mixed with 1000 μL R1, then incubated for 5 min. Furthermore, 250 μL R2 was added, and the absorbance value was read out at 1, 2, 3, and 4 min using a spectrophotometer at 365 nm. The equation determined the AST value:


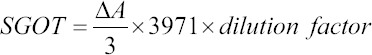


Where:

ΔA=The difference between the absorbance level at each measurement time [(A_1_-A_2_)+(A_2_-A_3_)+(A_3_-A_4_)]

A1=Absorbance value at the 1^st^ min

A2=Absorbance value at the 2^nd^ min

A3=Absorbance value at the 3^rd^ min

A4=Absorbance value at the 4^th^ min.

### Histopathological evaluation

The liver tissue samples were cut and fixed in neutral buffered formalin for 48 h. The tissues were processed manually using 70%, 80%, 90%, and 100% ethanol. The processed tissues were then embedded in a paraffin block and sectioned to a thickness of 5 μm using a rotary microtome. These sections were stained with hematoxylin and eosin. The slides were examined microscopically for histological changes, such as necrosis, swollen cells, and inflammation. The histological changes were examined under a light microscope (CX23 OLYMPUS Multimedia with Webcam Color High Definition Camera) at 40× (Shinjuku, Tokyo, Japan).

### Statistical analysis

The data were statistically analyzed using the Statistical Package for the Social Sciences (SPSS Inc., Chicago, IL, USA) 21.00 for Windows (α=0.05). The results were reported as the mean ± standard deviation (SD) of five replications.

## Results

### The antioxidant activity of OPME

Preliminary screening of OPME showed the presence of phenolics and flavonoids. The total phenolic level of okra OPME was 12.92 mg gallic acid/g sample, while the flavonoid level was 5.68 mg quercetin/g sample.

### Effect of OPME on antioxidant enzymes

The SOD level was significantly increased in the KN, P1, P2, P3, and P4 groups compared with that in the negative control (K-) group (p<0.05). The SOD test showed the lowest SOD level in the K-group (0.29±0.08 U/mL), while the highest SOD level was found in the KN group (0.73±0.02 U/mL). The SOD levels in the P1, P2, P3, and P4 groups were 0.47±0.05, 0.67±0.08, 0.67±0.05, and 0.71±0.07 U/mL, respectively.

Similar results were also found in the CAT levels. The CAT test showed that the negative control group (K-) had the lowest CAT level (9.11±0.27 U/mL), while the highest CAT level was found in the KN group (10.62±0.25 U/mL). The CAT levels in the P1, P2, P3, and P4 groups were 10.22±0.32, 10.07±0.31, 10.10±0.26, and 10.22±0.22 U/mL, respectively. Comparisons of both SOD and CAT levels among the groups are shown in Table-1.

**Table-1:** The effect of okra pods methanol extract on antioxidant enzymes, oxidant, and liver marker enzymes in sodium nitrite-induced hepatotoxicity.

Groups	CAT (U/mL)	SOD (U/mL)	NO (M)	MDA (μM)	ALT (U/L)	AST (U/L)
KN	10.62±0.25	0.73±0.02	0.23±0.02	15.60±1.41	31.14±1.14	21.77±1.42
K-	9.11±0.27	0.29±0.08	0.72±0.01	87.71±7.01	78.01±1.76	39.83±0.45
P1	10.22±0.32*	0.47±0.05**	0.22±0.02*	15.32±1.50*	33.36±3.05*	22.90±1.65*
P2	10.07±0.31*	0.67±0.08*	0.22±0.02*	16.10±1.12*	33.68±1.19*	21.91±0.90*
P3	10.10±0.26*	0.67±0.05*	0.24±0.02*	16.21±1.48*	35.29±0.88**	21.84±1.09*
P4	10.22±0.33*	0.71±0.07*	0.24±0.03*	16.43±1.31*	35.32±1.39**	21.10±0.93*

KN=Standard control group, K-=Negative control group (sodium nitrite induction 50 mg/kg BW), P1, P2, P3, and P4=Treatment group 1, 2, 3, and 4 (sodium nitrite induction 50 mg/kg BW and administration of OPME 50, 100, 200, and 400 mg/kg BW).

* significantly difference with K-group.

** significantly difference with K- and KN groups (a=0.05), ALT=Alanine aminotransferase, AST=Aspartate aminotransferase, SOD=Superoxide dismutase, CAT=Catalase, MAD=Malondialdehyde, NO: Nitric oxide

### Effect of OPME on oxidant level

Both NO and MDA levels were significantly increased in the negative control (K-) group compared with those in the KN, P1, P2, P3, and P4 groups (p<0.05). The NO and MDA oxidant levels in each group are shown in Table-1.

In terms of the measurements of NO level, the results showed that the negative control group (K-) had the highest NO level (0.72±0.01 M), while the lowest NO level was found in the P2 treatment group (0.22±0.02 M). The KN group had a NO level of 0.22±0.02 M, while these levels in the P1, P3, and P4 groups were 0.22±0.02, 0.24±0.02, and 0.24±0.03 M, respectively.

The MDA test showed that the negative control group (K-) had the highest MDA level (87.71±7.01 μM), while the lowest MDA level was found in treatment group P1 (15.32±1.50 μM). The MDA in the KN group was 15.60±1.41 μM that in P2 was 16.10±1.12 μM, that in P3 was 16.21±1.48 μM, and that in P4 was 16.43±1.31 μM.

### Effect of OPME on liver marker enzymes

Based on the ALT test, the negative control group (K-) had the highest ALT level of 78.01±1.76 U/L. Meanwhile, the KN group had the lowest ALT level of 31.14±1.14 U/L. The level in P1 was 33.36±3.05 U/L that in P2 was 33.68±1.19 U/L, that in P3 was 35.29±0.88 U/L, and that in P4 was 35.32±1.39 U/L (Table-1).

Based on the AST test, the negative control group (K-) had the highest AST level of 39.83±0.45 U/L, while the P4 treatment group had the lowest AST level of 21.10±0.93 U/L. The AST level in the KN group was 21.77±1.42 U/L that in the P1 group was 22.90±1.65 U/L, that in the P2 group was 21.91±0.90 U/L, and that in the P3 group was 21.84±1.09 U/L (Table-1).

### Effect of okra OPME on liver histopathology

**Figure-1:**
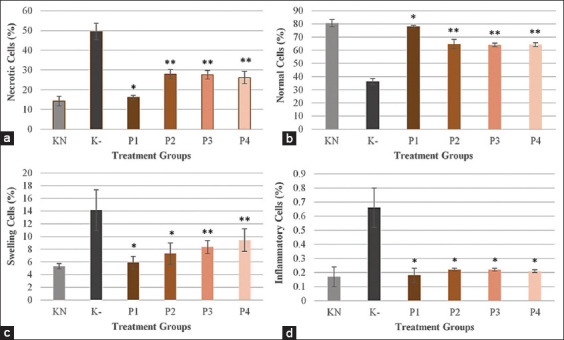
Effect of okra pods methanol extract (OPME) and sodium nitrite induction on the number of (a) necrotic cells, (b) normal cells, (c) swollen cells, and (d) number of inflammatory cells. KN: Standard control group, K-: Negative control group (sodium nitrite induction 50 mg/kg BW), P1, P2, P3, and P4: Treatment groups 1, 2, 3, and 4 (sodium nitrite induction 50 mg/kg BW and administration of OPME 50, 100, 200, and 400 mg/kg BW). *significantly difference with K- group, **significantly difference with K- and KN groups (α=0.05).

Data from the liver histological analysis include the number of necrotic cells, number of normal cells, proportion of swollen cells, number of inflammatory cells, and statistical test results, as shown in Figure-1. The treatment group that had the highest percentage of necrotic cells was the negative control group (K-) at 49.48±4.14%, while the standard control group (KN) had the lowest percentage of necrotic cells at 14.21±2.43%. Group P1 had 16.08±1.10% necrotic cells, P2 had 27.96±2.16%, P3 had 27.60±2.32%, and P4 had 26.19±3.16% (Figure-1a).

The treatment group with the highest proportion of normal cells was the standard control group (KN), with 80.47±2.75%, while the negative control group (K-) had the lowest percentage of normal cells at 36.36±2.24%. The corresponding value for P1 was 78.05±1.10% that for P2 was 64.73±3.50%, that for P3 was 64.07±1.40%, and that for P4 was 64.37±1.42% (Figure-1b).

The treatment group with the highest proportion of swollen cells was the negative control group (K-), at 14.17±3.20%, while the standard control group (KN) had the lowest proportion of swollen cellsat 5.31±0.41%. The corresponding value for group P1 was 5.87±0.01% that for P2 was 7.31±1.69%, that for P3 was 8.33±1.03%, and that for P4 was 9.43±1.76% (Figure-1c).

The treatment group with the highest number of inflammatory cells was the negative control group (K-), at 0.66±0.14%, while the standard control group (KN) had the lowest percentage at 0.17±0.07%. The percentage in the P1 group was 0.18±0.05% that in P2 was 0.22±0.01%, that in P3 was 0.22±0.01%, and that in P4 was 0.21±0.01% (Figure-1d).

## Discussion

Okra extract acts as an antioxidant. In this study, the potential of the extract is seen from its ability to stimulate the expression of antioxidant enzymes and inhibit oxidant compounds, which in turn can repair organ damage due to the presence of toxic substances. Okra extract contains various antioxidant compounds, such as flavonoids, quercetin, and phenolic acids, which can donate H^+^ atoms to ROS [11]. This is supported by the work of Beecher, who stated that quercetin in okra extract has several hydroxyl groups in its structure, which can donate H^+^ atoms to stabilize free-radical reactions [12]. Besides, okra extract also contains Vitamin C, which has been shown to have the ability to preserve MDA levels in mice like those in the standard group after exposure to free-radical compounds [13]. Vitamin C can restore lipid peroxidation levels to levels as high as in the control group [14]. The preliminary screening of OPME showed the presence of phenolic and flavonoid compounds. The total phenolic level of OPME was 12.92 mg gallic acid/g samples, and the flavonoid level was 5.68 mg quercetin/g sample.

Luo *et al*. [8] asserted that okra extract can induce the Nrf2-ARE pathway, a defense regulator that induces the expression of endogenous SOD and CAT antioxidant enzymes. SOD enzymes can bind superoxide and convert it to hydrogen peroxide, while CAT enzymes convert hydrogen peroxide into oxygen and water. An increase in the levels of SOD and CAT will reduce ROS levels. Decreasing ROS levels, in turn, prevent the formation of NO and inhibit it from turning into peroxynitrite (ONOO). In this study, it was proven that OPME can increase SOD and CAT, and also restore nitrite levels to normal. The results of the sodium dismutase test, in this study, show that the K-group had the lowest SOD level. In addition, the CAT test showed that the negative control group (K-) had the lowest CAT level. These conditions are similar to those in the NO test. This study showed that the K-group had the highest NO level, while the lowest nitrite level was found in the P2 treatment group (Table-1).

Decreased ROS levels prevent lipid peroxidation. When okra extract was administered to mice, PUFAs that initially bound to ROS (due to high levels) boundless when the amount of ROS decreased. This could inhibit the formation of MDA due to a decrease in PUFA peroxidation [15]. Therefore, the MDA level also declined along with the decrease of NO levels. The MDA test showed that the K-group had the highest MDA level, while the lowest MDA level was shown in the P1 treatment group (administered a low dose of 50 mg/kg BW of OPME) (Table-1).

In this research, MDA was also shown to play an essential role in the process of cell swelling. MDA is a product of lipid peroxidation. If lipid peroxidation occurs in the cell membrane, it damages this membrane. This, in turn, results in the disruption of ion transport because of the structural change of the Na^+^/K^+^ ATPase pump. Sodium ions, Ca^2+^, and water enter the cell, and ATP production in the mitochondria declines, after which the cells experience swelling. However, okra extract as an antioxidant can reduce MDA levels. Okra OPME reduces the likelihood of lipid peroxidation in cell membranes, so the structure of the Na^+^/K^+^ATPase pump is maintained. On maintaining the condition of the cell membrane, the structure of the Na^+^/K^+^ ATPase pump and Na^+^/Ca^2+^ channels will remain in good condition. The levels of Na^+^ and Ca^2+^ ions, and water in the cell will decrease, while the K^+^ levels will increase. Consequently, proportion of swollen cells will decrease [16]. The treatment group with the highest proportion of swollen cells was the K-group, while the P1, P2, P3, and P4 groups had lower percentages (Figure-1c).

Depleted cell swelling due to OPME induction causes a decrease of Ca^2+^ ions in the cell, thus preventing the transport of phospholipase, which causes plasma membrane damage [17]. Wallig and Evan [16], explained in their research that free radicals can cause mitochondrial dysfunction and the direct action of oxidant substances on membrane phospholipids also contributes to mitochondrial dysfunction. OPME acted as a free-radical scavenger that prevented the mitochondria from a condition called low-amplitude swelling, which reduces the production of ATP by mitochondria. It prevented the electrolytes from the outer compartment entering the inner compartment. If this process is inhibited, mitochondrial condensation will also be prevented, and the condition of high-amplitude mitochondrial swelling will not occur. The adenine nucleotide transporter will not form in the mitochondrial permeability transition, and organelles in the cell will be saved, preventing cell necrosis [16].

Wallig and Evan [16] also explained that condensation due to low-amplitude swelling could be solved if the toxic material that causes the condensation is removed, or other substances that can suppress the activity of the toxic material are removed. Therefore, it is possible that antioxidant okra extract plays a key role before or during the occurrence of low-amplitude mitochondrial swelling. In this study, the P1, P2, P3, and P4 groups had a lower percentage of necrotic cells than the K- group (Figure-1a).

**Figure-2:**
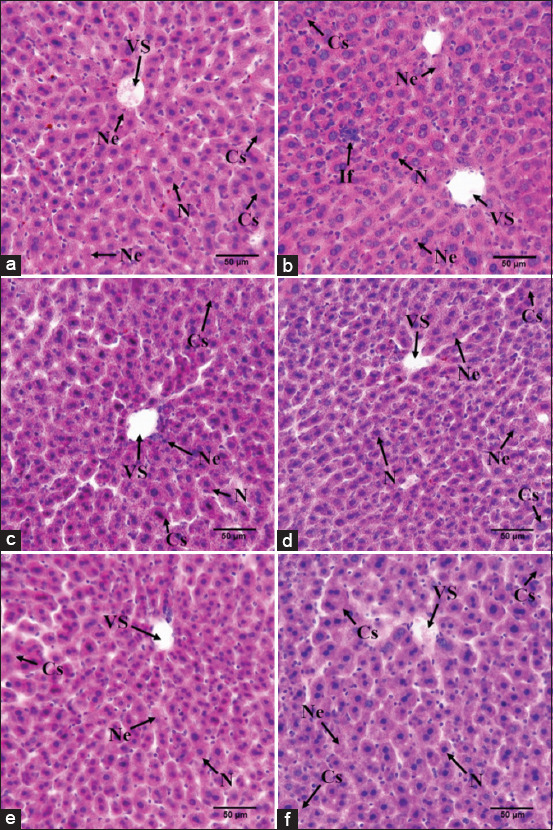
Effect of okra pods methanol extract and sodium nitrite administration on liver histological appearance (a) KN: Standard control group, (b) K-: Negative control group (sodium nitrite induction 50 mg/kg BW), (c) P1, (d) P2, (e) P3, and (f) P4: Treatment groups 1, 2, 3, and 4 (sodium nitrite induction 50 mg/kg BW and administration of OPME in 50, 100, 200, and 400 mg/kg BW). VS: Vena Centralis, N: Normal cells, Ne: Necrotic cells, B: Swollen cells.

The necrosis decreased due to the induction of OPME in the P1, P2, P3, and P4 groups. Accordingly, inflammation also decreased. When the number of cells undergoing necrosis decreases, this indicates that many cells are in good condition, in the sense that the cell membrane condition is still maintained, and cell organelles are not degraded. Therefore, damage-associated molecular patterns, chemo-attractants for the innate immune system, will not emerge from the cell. This is the cause of the absence of reactions from components of the innate immune system, such as neutrophils and macrophages. This, in turn, prevents the occurrence of an inflammatory reaction, thereby reducing the number of inflammatory cells. In this study, the highest number of inflammatory cells was shown by the K- group, (Figure-1d). The histological examination also showed better conditions in the P1, P2, P3, and P4 groups because the cells could be repaired to normal cells (Figure-2a-f).

Besides, OPME administration could reduce the number of necrotic cells in the P1, P2, P3, and P4 groups. In addition, it caused decreases in ALT and ASP levels, while also decreasing the number of cells undergoing necrosis. This means that the number of cells with ruptured membranes also decreased. This was the cause of the decreasing levels of both ALT and AST, ALT and AST are enzymes with high concentrations in cells, especially in hepatocytes. When the number of necrotic cells decreases, the levels of ALT and AST released into the bloodstream also decrease. In this study, although the ALT levels in the P3 and P4 groups differed significantly from that in the standard group (KN), the ALT levels in that group were still in the normal range, namely, 10-45 U/L (Table-1).

Dose-response relationships for OPME to the level of SOD, CAT, nitrite, and MDA, number of cells swelling (%), necrotic cells (%), number of inflammatory cells, and levels of ALT and AST are the new general curve model multiphasic. (Model enables description of a variety of dose-response cases where various phases are present). The curve model contains one stimulator and one inhibitor [18]. This curve illustrates that okra extract at low and moderate doses can provide an optimal response. At high doses, the response will be fixed (stationary). This proves that the OPME contained a compound that can alleviate oxidative stress conditions. It is possible that these compounds can be used as antioxidants to reduce ROS levels. However, at higher ROS levels, they have no reparative effect or can even act as inhibitors of the recovery from oxidative stress due to them inhibiting specific enzymes.

## Conclusion

This study showed that OPME containing flavonoid and phenolic compounds can increase SOD and CAT levels, and reduce excess levels of NO, MDA, ALT, and AST. The optimal dose of OPME as a treatment against oxidative stress induced by sodium nitrite was 50-100 mg/kg BW. This dose is optimal because it could improve SOD and CAT but degrade NO and MDA levels. It also reduced the proportion of swollen cells (%), necrotic cells (%), number of inflammatory cells (%), and also ALT and AST levels.

## Authors’ Contributions

SPAW and DW had the original idea for the study and carried out the design. ESS and BNDA collected the samples and analyzed the data. SPAW, ESS, MP, and DW drafted the manuscript. All authors revised the final manuscript and approved it.
